# Unobserved individual-level variation in cardiovascular mortality in birth cohorts born before and after 1945

**DOI:** 10.1186/s12872-026-05552-y

**Published:** 2026-02-04

**Authors:** Øyvind Næss, Huong Nguyen Thu

**Affiliations:** 1 Division for Mental and Physical Health, Norwegian Institute of Public Health, Oslo, Norway; 2 Centre for Fertility and Health, Norwegian Institute of Public Health, Oslo, Norway; 3 Institute of Health and Society, Faculty of Medicine, University of Oslo, PO Box 1130, Oslo, Blindern 0318 Norway

**Keywords:** Cardiovascular diseases, Mortality, Health behaviour, Survival, Cohort effect

## Abstract

**Aims:**

Cardiovascular disease (CVD) mortality has recently declined in many populations. It is not known if inequalities between individuals after accounting for risk factors have become more important. We investigated if the variation in risk due to unobserved factors – termed frailty - is larger in cohorts born after compared to before 1945.

**Methods:**

446,053 individuals born 1930–1945 and 1946–1960, participating in Norwegian health surveys (1974–1997) were followed for premature CVD mortality. We used proportional hazards models with Weibull baseline distributions to evaluate mortality after adjusting for CVD risk factors. Models included gamma frailty to quantify variation in risk due to unobserved factors between individuals within each birth cohort. Additionally, we calculated Gini coefficients and plotted Lorenz curves to compare mortality inequalities across cohorts.

**Results:**

Hazard ratio estimates for each CVD risk factor were similar in the two cohorts except for smoking. Frailty estimates after adjustment for all measured risk factors were 4.08 in the older cohort and 4.69 in the younger cohort, corresponding to nearly identical Gini coefficients of 0.79 and 0.77, respectively.

**Conclusion:**

Contrary to our hypothesis, we did not observe a substantial difference in unobserved individual-level variation in CVD mortality among those born after 1945. The nearly identical Gini coefficients indicate stable inequality over time. After accounting for established risk factors, CVD mortality remained highly concentrated, with roughly 80% of cumulative risk observed among approximately of 20% of individuals in the population.

**Supplementary Information:**

The online version contains supplementary material available at 10.1186/s12872-026-05552-y.

## Introduction

Many high-income countries have had a secular decline in premature cardiovascular disease (CVD) mortality in recent decades resulting in an upwards shift in the average age at which people first develop CVD [1]. Since the early 2000s, the rate of decline has slowed, now estimated at around 2% annually [2]. Mortality from CVD in high-income countries dropped significantly since 1960 – from 400 to 600 in 1960 to 100–200 in 2010 per 100,000 men and from 150 to 300 to fewer than 50 per 100,000 women [3]. Nordic countries, including Norway, have seen continuous and substantial declines [4]. Specifically, in Norway premature mortality due to ischemic heart disease fell markedly between 1980 and 2016, from 225 to 26 per 100,000 in men and from 63 to 9 per 100,000 in women.

Given these substantial population-level declines in mortality and traditional CVD risk factors, it is plausible that the distribution of underlying susceptibility to CVD, beyond observed risk factors, may also have shifted over time. Several reasons support this notion. First, individuals developing CVD today might differ fundamentally in their inherent susceptibility compared to previous generations, even at similar risk factor levels [1]. Moreover, the risks of coronary heart disease and stroke rise with age [5, 6], and biological aging is now delayed, particularly among socially advantaged groups [7]. Furthermore, improvements in survival following cardiovascular events, as well as reduced mortality in younger age groups, have likely altered the composition of the population at risk. Individuals surviving early cardiovascular events may face heightened risks for other non-communicable diseases, suggesting shared underlying susceptibility mechanisms [8]. Consequently, as conventional risk factors decline in prevalence, unobserved individual susceptibility might become relatively more important.

One way to operationalize these potential shifts in population-level susceptibility to CVD is through frailty survival models. Such models enable quantification of unobserved heterogeneity in mortality risks, facilitating comparisons across birth cohorts. Frailty models have previously revealed substantial variation in cancer risks, highlighting subpopulations with markedly elevated susceptibility, though their application to cardiovascular epidemiology remains limited. Prior studies suggest cancers with high heritability show larger inequalities in risk, reflecting the greater relative importance of genetic or biological susceptibility when environmental exposures diminish [9]. Similarly, for CVD, individuals at the highest polygenic risk percentiles exhibit risk levels comparable to monogenic mutations [10], indicating considerable inherent variation in susceptibility within populations.

We hypothesize that the decline in prevalence of observed cardiovascular risk factors in successive birth cohorts has led to an increased relative importance of unobserved individual-level variation (frailty) in cardiovascular mortality risk. Specifically, we expect that individuals born after 1945 exhibit greater heterogeneity in unmeasured susceptibility to premature cardiovascular mortality compared to those born earlier, reflecting a shifting balance from common environmental risks towards individual-level vulnerability.

## Methods

### Study population

From Norwegian population-based health surveys we selected participants born 1930–1945 and 1946–1960 who had not died before the time of the survey and who could be followed up until age 75. The surveys included the Counties Studies (1974-88) [11], the Age 40 Program (1985–1999) [12], and the Cohort of Norway (CONOR) (1994–2003) [13] with overall participation rate of 86%, 70% and 58%, respectively. Of 481,985 potential participants, we excluded 35,932 with missing information on risk factors. We further excluded individuals with inconsistent data and those who did not attend population census, had no registration, and had died, been censored, or emigrated before the survey, yielding a sample of 446,053 participants (Figure S1).

### Data linkage

Data from health surveys were linked to the Norwegian Cause of Death Registry, and the National Registry using the unique national personal identification number. If a participant attended several health surveys between ages 40–45 years, only the first survey was selected. Permission to be absolved from professional secrecy and linking of data was granted by the Regional Ethics Committee South-East (on May 25th, 2012, reference number 2012/872). The study complies with the Declaration of Helsinki.

### Cardiovascular risk factors

In all surveys, self-assessed questionnaires, clinical measures, and non-fasting blood sampling were collected with similar methodology and questionnaires [11–13]. Smoking status was collapsed into two categories: current smoker (yes) or never smoker or former smoker (no). Leisure time physical activity was harmonized into a four graded scale from sedentary (1) to hard physical (4). Blood pressure was initially measured manually using sphygmomanometers and the second of two measurements defined systolic blood pressure. Later, three automatic oscillometric measures were assessed and the average of the last two available measurements defined systolic blood pressure. Serum total cholesterol and triglycerides were initially measured by non-enzymatic, and later enzymatic method.

### Cardiovascular mortality

Data on underlying cause of death from CVD was obtained from the Norwegian Cause of Death Registry through 2020 (ICD-8: 390-444.1, 444.3–458, 782.4, ICD-9: 390–459, ICD-10: I00-I99). The participants were followed prospectively from the time of health examination up to death, emigration, or age 75.

### Statistical methods

We considered two birth cohorts, those who were born during 1930–1945 and 1946–1960. For each birth cohort *i* (i = 1, 2), we estimated a frailty model in the form of a conditional hazard function:

1 $${h_{\mathrm{i}}}({\mathrm{t}}|{{\mathrm{Z}}_{\mathrm{i}}}){\text{ }}={\text{ }}{h_0}({\mathrm{t}}){{\mathrm{Z}}_{\mathrm{i}}}{\mathrm{exp}}(\boldsymbol{\beta}{\mathbf{x}_{\mathrm{i}}})$$

Z_i_ is a measurement of heterogeneity in the birth cohort *i*. Analysis time was measured in years (number of days divided by 365.25) from the date of survey until date of death, date of emigration or censoring. *h*_*i*_*(t|Z*_*i*_*)* was the conditional hazard of the individuals who belonged to birth cohort *i*. The baseline hazard function at time *t* was *h*_*0*_*(t)*. We denoted ***x***_***i***_ as a row vector of covariates and its corresponding coefficient vector as β. We assumed the baseline hazard function to follow a Weibull distribution [14], *h*_*0*_*(t) = pt*^*p−1*^. The shape parameter *p* allows the density to take a variety of shapes. The shape may be influenced by birth cohorts and vary. The choice of Weibull distribution is based on the nature of the CVD mortality data and the underlying biological process relating to the CVD. We further assumed a Gamma distributed frailty with parameter θ [15]. Parameter θ represents the variance of the unobserved individual-level frailty term, modelled using a gamma distribution with mean 1 and variance θ. This distribution captures unmeasured heterogeneity in baseline hazard across individuals.

We added sex and risk factors (systolic blood pressure (mm Hg), total cholesterol (mmol/l), triglycerides (mmol/l), current smoking status (yes/no) and physical inactivity) (1–4). Our frailty models can be given by

2 $${h_{\mathrm{i}}}({\mathrm{t}}|{{\mathrm{Z}}_{\mathrm{i}}}){\text{ }}={\text{ }}{{\mathrm{Z}}_{\mathrm{i}}}{\mathrm{p}}{{\mathrm{t}}^{p - 1}}{\mathrm{exp}}({\beta _0}+{\beta _1}{{\mathrm{x}}_{{\mathrm{i1}}}}+{\beta _2}{{\mathrm{x}}_{{\mathrm{i2}}}}+...+{\beta _j}{{\mathrm{x}}_{{\mathrm{ij}}}}+...+{\beta _6}{{\mathrm{x}}_{{\mathrm{i6}}}})$$

where *x*_*ij*_ is the covariate *j*, where j = 1,.,6. for the individuals belonging to birth cohort *i* (the covariate *j*, *x*_*i*j_ is for the individuals who belonged to cohort *i)*. We calculated the Gini indices for the frailty distributions in the two birth cohorts, using the acid-package in R. The Gini coefficients represent inequality of frailty for different birth cohorts [16]. The acid package in R includes a function to compute the Gini coefficient for a gamma distribution based on the shape parameter (shape, q). Specifically, the following formula,

$$\mathrm{G}\;=\;[2\;\times\Gamma(\mathrm{q}\;+\;1/2)]\;/\;[\surd\pi\;\times\;\Gamma(\mathrm{q})]\;-\;1$$

assuming the gamma distribution is standardized with a mean of 1 and variance equal to 1/q, as is typical for frailty terms in survival models [17, 18]. A Gini coefficient of zero expresses perfect equality, i.e., no unobserved variation between individuals. A Gini coefficient of one indicates maximal inequality. Lorenz curves were plotted to provide a visualisation of inequalities in individual risk according to birth cohorts at population level.

We included age at the start of follow-up (i.e., age at the time of the health examination) as a covariate in a sensitivity analysis. We also investigated if the main effects of each risk factor differed by birth cohort in additional analysis including interaction terms.

## Results

The oldest cohort (born before 1945) represented a smaller proportion (8%) of the study population compared to the younger cohort (born after 1945) (Table 1). The cohorts differed notably in age at the start of follow-up: the older cohort had a mean age of 58.6 years (SD 5.8) and median of 59.4 years (IQR 54.4–61.6), whereas the younger cohort had a mean age of 41.6 years (SD 2.4) and median of 41.4 years (IQR 40.6–42.3). Follow-up duration also differed between cohorts, averaging 15.4 years (SD 6.2) with a median of 15.0 years in the older group, compared to 25.9 years (SD 4.9) with a median of 26.2 years in the younger group.

**Table 1:** Descriptive statistics according to categories of birth cohorts among participants in Norwegian health surveys (n = 446,053)

	Birth cohorts		
	Born before 1945	Born after 1945	Total
All (%)	37,062 (8.31)	408,991 (91.69)	446,053 (100)
Age at start of follow-up	58.59 (5.83)	41.63 (2.39)	43.05 (5.47)
Follow-up time in years	15.38 (6.21)	25.90 (4.89)	25.03 (5.80)
CVD deaths (%)	3736 (10.08)	7204 (1.76)	10,940 (2.45)
Sex (male), n (%)	19,795 (53.41)	194,533 (47.56)	214,328 (48.05)
Systolic blood pressure, mm Hg	140.21 (20.55)	128.75 (14.90)	129.71 (15.77)
Total cholesterol, mmol/l	6.29 (1.22)	5.61 (1.08)	5.67 (1.10)
Triglycerides, mmol/l	1.80 (1.15)	1.72 (1.22)	1.73 (1.22)
Current smoking status, n (%)	11,652 (31.44)	162,636 (39.77)	174,288 (39.07)
Physical active (1–4)	1.84 (0.85)	2.04 (0.79)	2.02 (0.80)

Presented as mean (standard deviation) or count (percentages)

The levels of cardiovascular risk factors differed between the two cohorts. Systolic blood pressure, total cholesterol, and triglyceride levels appeared to decline in the younger cohort, while the proportion of physically inactive individuals was higher. The proportion of current smokers was 31% in the older cohort and 40% in the younger. Over a total follow-up of 6,488,891 person-years (mean 18 years; range 1 day to 21 years), 16,665 individuals (4.6%) died prematurely from any cause, 3,871 (1.1%) died prematurely from CVD, and 0.5% emigrated. CVD mortality was lower in women than in men.

Table 2 presents hazard ratio (HR) estimates and corresponding 95% confidence intervals (CIs) for CVD mortality, based on Cox proportional hazards regression models. In Model 1, estimates are adjusted for sex, with each risk factor entered separately. The HR for current smoking was notably lower in the older cohort (HR 1.64, 95% CI: 1.54–1.75) compared to the younger cohort (HR 2.80, 95% CI: 2.66–2.93). Estimates for other risk factors differed less markedly between cohorts. In the mutually adjusted Model 2, results remained largely unchanged. For current smoking, the HR was 1.67 in the older cohort and 2.75 in the younger.

**Table 2:** HRs and 95% CIs for CVD mortality according to established risk factors of CVD among participants in Norwegian health surveys (n = 446,053), estimated using Cox regression

	Birth cohorts					
	Born before 1945			Born after 1945	Total	
Model 1						
Systolic blood pressure, mm Hg		1.02 (1.02 – 1.02)	1.03 (1.03 – 1.03)			1.04 (1.04 – 1.04)
Total cholesterol, mmol/l		1.09 (1.09 – 1.10)	1.09 (1.09 – 1.10)			1.09 (1.09 – 1.10)
Triglycerides, mmol/l		1.10 (1.08 – 1.11)	1.08 (1.08 – 1.09)			1.08 (1.08 – 1.08)
Current smoking status		1.64 (1.54 – 1.75)	2.80 (2.66 – 2.93)			2.08 (2.00 – 2.16)
Physical inactive		0.80 (0.77 – 0.84)	0. 80 (0.78 – 0.82)			0.73 (0.71 – 0.75)
Model 2						
Systolic blood pressure, mm Hg		1.02 (1.02 – 1.02)	1.03 (1.03 – 1.03)			1.04 (1.03 – 1.04)
Total cholesterol, mmol/l		1.03 (1.00 – 1.06)	1.09 (1.09 – 1.10)			1.10 (1.09 – 1.10)
Triglycerides, mmol/l		1.06 (1.04 – 1.08)	1.04 (1.04 – 1.05)			1.03 (1.02 – 1.04)
Current smoking status		1.67 (1.60 – 1.79)	2.75 (2.62 – 2.89)			2.06 (1.99 – 2.14)
Physical inactive		0.84 (0.81 – 0.88)	0.88 (0.86 – 0.91)			0.80 (0.78 – 0.81)

*Abbreviations: CVD* cardiovascular mortality, *HR* hazard ratio, *CI* confidence interval

Model 1: adjusted for sex. Model 2: adjusted for sex and the other CVD risk factors.  Numbers in parentheses are the corresponding 95% confidence intervals

In the regression models that included a frailty variance parameter (Table 3), the estimated frailty variance (θ) was similar between the two cohorts: 4.08 (95% CI: 2.95–5.65) in the older cohort and 4.69 (95% CI: 3.23–6.82) in the younger cohort. For ease of interpretation, these correspond to Gini coefficients of 0.77 and 0.79, respectively. In the main model (Table 3), the shape parameters ranged from 4.08 to 6.69, and the scale parameters ranged from 3.87 to 4.69. In the age-adjusted sensitivity analysis (Supplementary Table 1), the shape estimates were similar, but the scale parameters were lower—particularly in the total population, where the scale parameter was 0.48.

**Table 3:** HRs and 95% CIs for CVD mortality according to established risk factors of CVD among participants in Norwegian health surveys (n = 446,053), stratified by birth cohort estimated with Weibull baseline hazard distribution and Gamma frailty distribution

	Born before 1945	Born after 1945	Total
Sex (male)	2.93 (2.56–3.35)	2.07 (1.95–2.20)	2.05 (1.95–2.16)
Systolic blood pressure, mm Hg	1.02 (1.02–1.03)	1.03 (1.03–1.03)	1.04 (1.04 − 1.04)
Total cholesterol, mmol/l	1.04 (1.00–1.08)	1.31 (1.28–1.34)	1.37 (1.34–1.39)
Triglycerides, mmol/l	1.15 (1.09–1.20)	1.07 (1.05– 1.09)	1.01 (1.00–1.03)
Current smoking status	2.06 (1.84– 2.30)	2.90 (2.73 − 3.08)	2.18 (2.08–2.28)
Physical inactive	0.78 (0.74–0.83)	0.89 (0.86– 0.92)	0.79 (0.77–0.81)
Log(constant)	-33.1 (-36.1 – -30.0)	-28.9 (-29.8 – -27.8)	-27.7-28.5 – -26.9)
$\:\widehat{p}$	6.69 (6.02–7.44)	4.49 (4.30–4.70)	4.08 (3.92–4.25)
$\:\widehat{\theta\:}$	4.08 (2.95–5.65)	4.69 (3.23–6.82)	3.87 (3.03–4.94)
Chi-squared (p-value)	72.52 (< 0.001)	319.17 (< 0.001)	613.91 (< 0.001)

Numbers in parentheses are the corresponding 95% confidence intervals. Chi-squared is the likelihood-ratio (LR) test statistic of H_0_ θ = 0

Abbreviations

In supplementary analyses, adjusting for age at the start of follow-up gave consistent results with the main findings in Table 3 (Supplementary Table 1). This substantially reduced unobserved heterogeneity, particularly in the older cohort. Interaction tests indicated that the associations between several cardiovascular risk factors and CVD mortality differed significantly by birth cohort (Supplementary Table 2). Significant interactions with birth cohort were observed for age at start of follow-up, systolic blood pressure, total cholesterol, triglycerides, current smoking status, and physical inactivity (all *p* < 0.01). No significant interaction was found for sex. We also examined proportional hazards assumptions using frailty-adjusted Schoenfeld residuals. Plots of residuals against time showed no systematic deviations from proportionality.

## Discussion

### Main findings

We investigated, using large-scale pooled health surveys, whether unobserved individual-level variation (frailty) in premature cardiovascular mortality (defined as death before age 75) has increased in more recent birth cohorts, in the context of declining population-level CVD risk factors and mortality. Contrary to our hypothesis, frailty variance and Gini coefficients were nearly identical between cohorts (0.77 vs. 0.79), indicating stable levels of unmeasured risk heterogeneity. The distribution of risk remained highly unequal: approximately 20% of individuals accounted for 80% of the cumulative mortality risk. These findings suggest that, despite changes in overall CVD burden and risk factor prevalence, the relative contribution of unobserved susceptibility has remained constant.

### Strengths and limitations

This study has several notable strengths. First, it leverages a large, population-based sample of over 440,000 individuals drawn from nationally representative Norwegian health surveys, with long-term follow-up for mortality. This enables robust estimation of premature CVD mortality across two distinct birth cohorts. Second, we employed frailty survival models alongside Gini coefficients and Lorenz curves—a novel approach for assessing inequality in unobserved risk. Third, the large-scale survey data included detailed information on key cardiovascular risk factors, allowing us to examine unobserved variation in risk more directly.

However, several limitations should be considered. First, cardiovascular risk factors were measured at a single time point, limiting our ability to account for changes in risk profiles over the life course or during follow-up. In addition, many covariates were self-reported, making them susceptible to recall bias or misclassification. This issue is particularly relevant given the historical context of the data collection and may have introduced variability in reporting between birth cohorts. Consequently, frailty variance may partly reflect measurement error, temporal instability of risk factors, and unobserved behavioral changes, which may differ across birth cohorts. Frailty should therefore be interpreted as capturing both stable unmeasured susceptibility and residual time-varying or mismeasured risk. Secondly, although we harmonized data across multiple survey waves, differences in participation rates and follow-up time may have introduced residual heterogeneity not fully accounted for in the models. For instance, the younger cohort was followed from their forties and fifties, whereas the older cohort entered the study in their late fifties. This discrepancy could introduce bias as differences in age at baseline and follow-up between cohorts may affect frailty estimation. Individuals with higher unobserved risk are less likely to survive studying entry, particularly in the older cohort. This may have attenuated frailty due to selective survival. However, as most CVD events occur at older ages, the impact is likely limited. In sensitivity analyses adjusting for age at start of follow-up, the estimated frailty variance was more attenuated in the older cohort born before 1945, likely because they were assessed at older ages. This suggests that having comparable follow-up during older age as we do (avoiding right truncation) is more influential than differences in age at study entry (i.e., left truncation). It is nevertheless difficult to fully assess the impact of this potential bias.

Thirdly, socioeconomic position, education, and income are important determinants of cardiovascular risk but were not included due to limited comparability across cohorts in pooled analyses. Their omission may have resulted in residual confounding that is absorbed by the frailty term. Consequently, stable frailty variance across cohorts may partly reflect persistent unmeasured social stratification, which limits causal interpretation regarding generational stability in biological susceptibility.

Fourth, participation rates declined across the successive surveys, which may have introduced selection bias. If non-participation is correlated with unobserved frailty such that individuals with higher underlying cardiovascular risk are less likely to attend, then frailty variance may be underestimated. Moreover, cohort differences in participation could mask true differences in unobserved heterogeneity. The direction and magnitude of this bias cannot be quantified with the available data.

Additionally, as noted by Gómez (2002) [15], frailty variance estimates may be sensitive to the specification of the baseline hazard, which could influence the results. Our findings depend on the Weibull baseline hazard and gamma-distributed frailty assumptions. Alternative choices could yield different absolute levels of frailty variance. Accordingly, our conclusions emphasize relative patterns and consistency across complementary measures including frailty variance, Gini coefficients, and Lorenz curves rather than precise estimates of unobserved risk.

Fitting frailty models requires substantial statistical power. The oldest birth cohort comprised a relatively small proportion of the study population (less than 10%), limiting our ability to model it separately with sufficient precision. Initially, we planned to divide the sample into three birth cohorts—those born in the 1930s, 1940s, and 1950s—based on the same categorization used in a related study on the mediating role of risk factors in educational inequalities in CVD mortality [19]. However, power was insufficient to fit frailty models across all three groups. We therefore combined participants into two cohorts, born before and after 1945, aligning with a period marked by a four- to five-fold decline in CVD mortality (from 1970 to 2020) as the population entered middle age. The younger cohort was examined at a lower age, which may have contributed to higher estimated relative risks for some risk factors—particularly smoking. This pattern was evident for current smoking but not for other risk factors. The relative effect of smoking on CVD mortality has been shown to be stronger at younger ages, as baseline CVD risk is lower, resulting in a greater proportional increase in risk due to smoking [20]. In the older cohort, selective survival may also have influenced the results, as individuals particularly susceptible to smoking-related harms may not have survived into their late fifties. Historical data indicate that daily smoking prevalence was higher among men in the oldest birth cohorts and that the decline in smoking was more pronounced in younger age groups [21]. We did not have data on smoking cessation after baseline, so we could not account for changes in smoking behaviour during the follow-up period. Finally, changes in cause of death coding and the increasing registration of dementia and other causes as an underlying cause may have contributed to declining CVD mortality [22], but such competing causes mainly affect older ages. As shown by Berry et al. [23], this influence becomes more pronounced after age 75, above the upper age limit in our analysis.

### Interpretation

Assuming a Weibull distribution for the baseline hazard is reasonable in this context [14], as individual-level CVD risk typically increases with age due to physiological changes in the cardiovascular system. While risk estimates for some CVD risk factors may attenuate in older age groups [21], others—such as obesity and diabetes—are more strongly associated with aging [24]. Age-related structural and functional changes, including myocardial thickening, arterial stiffening, and reduced cardiac reserve, contribute to elevated risks of atherosclerosis, hypertension, myocardial infarction, and stroke [5, 6]. For example, studies have shown that carotid intima-media thickness can increase two- to threefold between ages 20 and 90. In our analysis, we assumed a gamma-distributed frailty term, which is commonly used in survival analysis due to its mathematical tractability and its ability to capture unobserved heterogeneity in age-related outcomes like CVD mortality [25].

The frailty parameter is a useful tool for quantifying unobserved variation in risk but may be less intuitive for some readers, given its limited use in epidemiological research and its ability to capture multiple sources of residual risk. We plotted Lorenz curves for each birth cohort, accompanied by Gini coefficients as summary measures of inequality. The Gini index is rarely used in epidemiology [16], but it is a useful tool for comparing inequality across populations of different sizes. It is based on the relative mean absolute difference in risk between individuals and ranges from 0 (perfect equality) to 1 (maximum inequality). The Lorenz curve is defined by a function *L (F)*, where *F* represents the cumulative proportion of the population (x-axis), and *L* the cumulative proportion of unobserved CVD mortality risk (y-axis). In a perfectly equal distribution, 50% of the population would carry exactly 50% of the risk, resulting in a straight diagonal line. The Gini index quantifies the deviation from this line, with greater curvature indicating greater inequality. In our analysis, the Gini coefficients were 0.77 and 0.79 for the two cohorts, indicating substantial inequality in unobserved CVD risk. This level of inequality is comparable to, or even greater than, what has been reported for many types of cancer [26]. In practical terms, the Lorenz curve showed that approximately 80% of the cumulative risk was concentrated in just 20% of the population.

There has been considerable interest in understanding the extent to which established cardiovascular disease (CVD) risk factors explain differences in outcomes between populations or social groups, as this has important implications for prevention strategies and efforts to reduce health inequalities [27–29]. A key question is whether interventions aimed at lowering these risk factors will have similar effects across different populations. Previous evidence suggests that modifiable cardiometabolic and behavioral risk factors explain a substantial portion of inequalities, particularly when measured longitudinally and using absolute measures of social group differences [19, 27]. Our findings support this evidence using a different approach. Specifically, the frailty variance in models without adjustment for risk factors (frailty variance: 34.2, not tabulated) was far greater than the difference in frailty variance between birth cohorts, indicating that unmeasured variation is substantially reduced when key risk factors are accounted for. However, even after adjustment, a large subpopulation—about 20%—still accounted for 80% of the residual risk in both cohorts, highlighting the persistence of substantial individual-level heterogeneity.

When comparing populations or subgroups, the relative contribution of risk factors such as smoking may dominate CVD risk in groups where smoking is highly prevalent. In contrast, in groups where smoking is less common, other factors—including unobserved or genetic ones—may become proportionally more important. This reflects Geoffrey Rose’s observation that if smoking were ubiquitous, differences in lung cancer incidence would be more strongly attributed to other factors, such as asbestos exposure or genetic factors [30]. Nonetheless, interventions targeting smoking would still yield the largest absolute reduction in risk and in health inequalities. In this study, we hypothesized that the substantial decline in CVD mortality across successive birth cohorts would lead to increased relative importance of underlying individual susceptibility—such as genetic factors—as modifiable risk factors became less prevalent. This hypothesis was motivated by recent studies showing that polygenic risk to CVD and adherence to healthy lifestyle may have strong population-level influence and have comparable effect to rare monogenic disorders such as familial hypercholesterolemia [10, 31, 32]. However, our findings did not support this hypothesis. It is possible that the results reflect model misspecification or limitations in available covariates. Further studies incorporating more detailed measures of genetic, behavioral, and environmental factors are needed to better assess changes in unobserved susceptibility over time.

Our findings suggest that unobserved individual-level variation in cardiovascular mortality has remained stable across cohorts born before and after 1945. Whether this pattern persists in later-born cohorts is uncertain. Among those born after 1960, continued declines in cardiovascular mortality have coincided with lower smoking, blood pressure, and cholesterol levels, but higher obesity and physical inactivity [1]. Future studies should assess whether such shifts modify the balance between observed and unobserved determinants of cardiovascular mortality.

From a public health perspective, it is important to understand how population-level changes affect the age group considered at risk for premature CVD, as this group is a key target for preventive efforts. Preventing premature CVD cases can substantially impact overall population health and reduce the burden of disease, given that CVD shares risk factors with many other non-communicable diseases [33]. The WHO Global Action Plan for the Prevention and Control of Noncommunicable Diseases calls on member states to reduce mortality from major NCDs among people aged 30–70 years, with a focus on addressing a few key modifiable risk factors.

## Conclusions

**Fig. 1:**
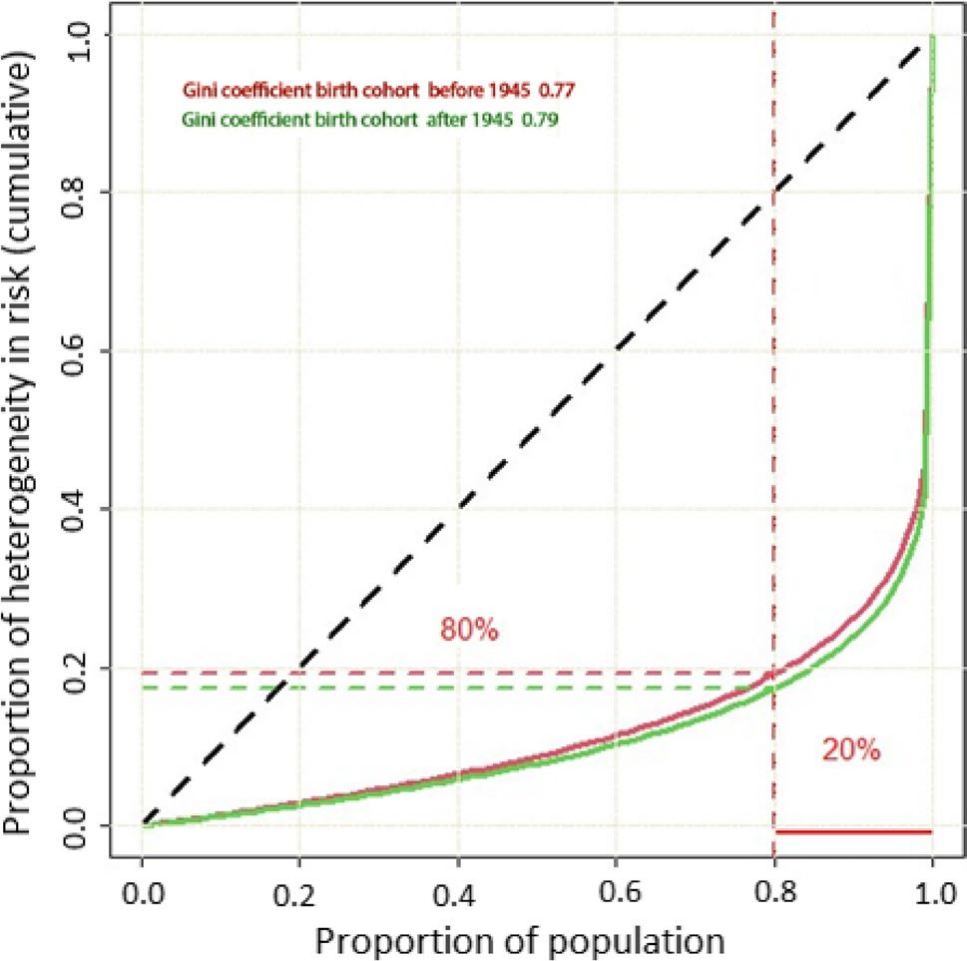
Plotted Lorentz curves in two birth cohorts born before (red) and after (green) 1945 with the corresponding Gini coefficients (proportion under the curve deviating from the diagonal). The horizontal x-axis shows the cumulative proportion of the population, and the vertical y-axis shows the proportion of unobserved CVD mortality in the population

Contrary to our initial hypothesis, we did not observe a substantial increase in unobserved individual-level variation in premature CVD mortality in the later-born cohort. The Gini coefficients were nearly identical between the two birth cohorts (0.77 vs. 0.79), indicating that the degree of inequality in unmeasured risk remained stable despite significant declines in overall CVD mortality and changes in the distribution of observed risk factors. This suggests that the relative importance of unmeasured individual frailty has not increased in more recent cohorts. The distribution of risk remained highly unequal, with approximately 80% of cumulative risk concentrated in just 20% of the population (Fig. 1).

## Supplementary Information

Supplementary Material 1.

Supplementary Material 2.

## Data Availability

Data is stored on a secure server at the University of Oslo. The corresponding author can give access to researchers who want to analyse data.
