# Supplementary material for: Unobserved individual-level variation in cardiovascular mortality in birth cohorts born before and after 1945
Source: BMC Cardiovasc Disord. 2026 Feb 4;26:197. doi: 10.1186/s12872-026-05552-y (PMC12958771; doi:10.1186/s12872-026-05552-y)
Supplement: Supplementary file 1 — Supplementary Material 1. [file 12872_2026_5552_MOESM1_ESM.docx]

**Supplementary material**

**Target population**

(Norwegian adult population)

**Source population**

Age 40 Program

1985-1999

n=417,336

19

(n = )

Cohort of Norway

1994-2003

n=172,798

19

(n = )

Counties Study

1974–1988

n=94,022

19

(n = )

**Total number of observations**

n=684,156

Overlapping participants (n=118,592)

**Individuals**

n= 565,564

Excluded and/or could not determine the birth cohorts (n= 83,579)

**Total number of eligible unique participants**

n= 481,985

**Excluded**

Did not attend the census (n=437)

Missing on registration and died before survey (n=4)

Censored before survey (n= 2,139)

Emigrated before survey (n= 21)

Inconsistent follow-up data (n= 1,275)

Missing CVD risk factors

Current smoking (n= 26,810)

Physical activity (n= 6,403)

Triglycerides (n= 519)

Total cholesterol (n= 2)

Systolic blood pressure (n= 461)

**Eligible participants with data on birth cohorts**

**(n= 446,053)**

Supplementary Figure 1. Flow chart on inclusion and exclusion
