# Supplementary material for: Unobserved individual-level variation in cardiovascular mortality in birth cohorts born before and after 1945
Source: BMC Cardiovasc Disord. 2026 Feb 4;26:197. doi: 10.1186/s12872-026-05552-y (PMC12958771; doi:10.1186/s12872-026-05552-y)
Supplement: Supplementary file 2 — Supplementary Material 2. [file 12872_2026_5552_MOESM2_ESM.docx]

| **Supplementary Table 1.** HRs and 95% CIs for CVD mortality according to established risk factors of CVD among participants in Norwegian health surveys (n = 446,053), stratified by birth cohort estimated with Weibull baseline hazard distribution and Gamma frailty distribution. | | | |
| --- | --- | --- | --- |
|  | **Born before 1945** | **Born after 1945** | **Total** |
| Age at start of follow-up | 1.19 (1.19 – 1.20) | 1.03 (1.02 – 1.04) | 1.14 (1.14 – 1.14) |
| Sex (male) | 1.95 (1.82 – 2.09) | 2.08 (1.96 – 2.21) | 2.16 (2.07 – 2.25) |
| Systolic blood pressure, mm Hg | 1.01 (1.01 – 1.02) | 1.03 (1.03 – 1.03) | 1.02 (1.02 – 1.02) |
| Total cholesterol, mmol/l | 1.08 (1.06 – 1.10) | 1.31 (1.27 – 1.34) | 1.21 (1.19 – 1.23) |
| Triglycerides, mmol/l | 1.07 (1.05 – 1.09) | 1.07 (1.05 – 1.09) | 1.06 (1.05 – 1.08) |
| Current smoking status | 1.94 (1.82 – 2.08) | 2.92 (2.74 – 3.10) | 2.47 (2.37 – 2.57) |
| Physical inactive | 0.84 (0.81 – 0.88) | 0.89 (0.86 – 0.92) | 0.89 (0.87 – 0.91) |
| Log(constant) | -43.3 (-45.0 – -41.8) | -30.0 (-31.4 – -28.8) | -32.87 (-33.54 – -32.21) |
| $\hat{p}$ | 7.01 (6.69 – 7.35) | 4.47 (4.27 – 4.67) | 4.53 (4.39 – 4.68) |
| $\hat{\theta}$ | 0.00 (0.00 – 0.00) | 4.61 (3.18 – 6.68) | 0.48 (0.36 – 0.64) |
| Chi-squared (p-value) | 0.00 (0.49) | 310.16 (<0.001) | 170.60 (<0.001) |

Note: numbers in parentheses are the corresponding 95% confidence intervals. Chi-squared is the likelihood-ratio (LR) test statistic of H­_0_ θ = 0.

| **Supplementary Table 2.** HRs and 95% CIs for CVD mortality from joint model with birth cohort as a covariate | |
| --- | --- |
| **Covariates** | **HR (95% CI)** |
| Age at start of follow-up | 1.12 (1.12–1.13) |
| Sex (male) | 2.17 (2.08–2.27) |
| Systolic blood pressure, mm Hg | 1.02 (1.02–1.02) |
| Total cholesterol, mmol/l | 1.20 (1.18–1.22) |
| Triglycerides, mmol/l | 1.07 (1.05–1.08) |
| Current smoking status | 2.47 (2.37–2.58) |
| Physical inactivity | 0.89 (0.87–0.91) |
| Cohort 2 (vs Cohort 1) | 0.89 (0.87–0.91) |
| Interaction Test | Chi-squared (p-value) |
| Cohort × Age at start | 494.93 (p=0.0000) |
| Cohort × Sex | 0.27 (p=0.6022) |
| Cohort × Systolic blood pressure | 183.78 (p=0.0000) |
| Cohort × Total cholesterol | 91.46 (p=0.0000) |
| Cohort × Triglycerides | 9.73 (p=0.0018) |
| Cohort × Current smoking status | 51.32 (p=0.0000) |
| Cohort × Physical inactivity | 49.78 (p=0.0000) |
| Note: Cohort 1 is cohort born before 1945, and cohort 2 is the one after 1945. Interaction tests if there's evidence of effect modification by birth cohort for each of CVD risk factor. If p>0.05, there was no significant interaction (the association between CVD mortality and corresponding CVD risk factor does not differ by cohort group). | |
